# Factors at multiple scales drive parasite community structure

**DOI:** 10.1111/1365-2656.13853

**Published:** 2022-11-29

**Authors:** Joshua I. Brian, David C. Aldridge

**Affiliations:** ^1^ Aquatic Ecology Group, The David Attenborough Building, Department of Zoology University of Cambridge Cambridge UK; ^2^ Department of Geography, Bush House NE, King's College London London UK

**Keywords:** community assembly, competition, freshwater mussel, infection, Markov Random Fields model, nestedness, null model, turnover, *β*‐diversity

## Abstract

1. Understanding how ecological communities are assembled remains a key goal of ecosystem ecology. Because communities are hierarchical, factors acting at multiple scales can contribute to patterns of community structure. Parasites provide a natural system to explore this idea, as they exist as discrete communities within host individuals, which are themselves part of a community and metacommunity.
2. We aimed to understand the relative contribution of multi‐scale drivers in parasite community assembly and assess how patterns at one level may mask those occurring at another. Specifically, we wanted to disentangle patterns caused by passive sampling from those determined by ecological drivers, and how these vary with scale.
3. We applied a Markov Random Fields model and assessed measures of *β*‐diversity and nestedness for 420 replicate parasite infracommunities (parasite assemblages in host individuals) across two freshwater mussel host species, three sites and two time periods, comparing our results to simulations from four different ecologically relevant null models.
4. We showed that *β*‐diversity between sites (explaining 25% of variation in parasite distribution) and host species (41%) is greater than expected, and *β*‐diversity between individual hosts is smaller than expected, even after accounting for parasite prevalence and characteristics of host individuals. Furthermore, parasite communities were significantly less nested than expected once parasite prevalence and host characteristics were both accounted for, but more nested than expected otherwise, suggesting a degree of modularity at the within‐host level that is masked if underlying host and parasite characteristics are not taken into account. The Markov Random Fields model provided evidence for possible competitive within‐host parasite interactions, providing a mechanism for the observed infracommunity modularity.
5. An integrative approach that examines factors at multiple scales is necessary to understand the composition of ecological communities. Furthermore, patterns at one level can alter the interpretation of ecologically important drivers at another if variation at higher scales is not accounted for.

## Graphical abstract

Beta‐diversity and nestedness may vary with ecological scale, which can reveal important drivers of community assembly and structure. Using parasite communities, the authors demonstrate that failing to account for patterns at one level alters the interpretation of ecological drivers at another.

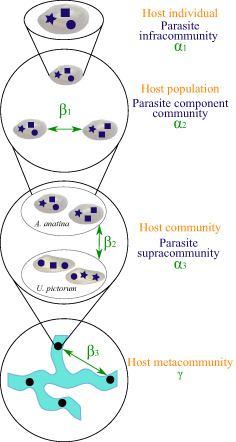

## 1 INTRODUCTION

Understanding the assembly of communities remains an important goal for managing them in the present and predicting how they will change in the future. This goal is complicated by the inherently hierarchical nature of ecological communities, with individuals existing as members of populations, communities and metacommunities. Different processes can act at different levels of the hierarchy (Bolnick et al., 2020), leading to patterns that differ depending on what scale the system is observed at (e.g. Chase et al., 2019). It is therefore desirable to understand how processes at different scales interact to facilitate a more mechanistic understanding of community structure (Thompson et al., 2020).

A significant body of work exists concerning community assembly across scales. For example, the metacommunity approach centres on a set of linked local communities (Leibold et al., 2004), while the principle of centrifugal community organisation emphasises the role of primary and secondary habitat selection when species arrive from a regional species pool, with that selection driven by local densities of different species (Rosenzweig & Abramsky, 1986). Both lean heavily on the strength of dispersal in determining community assembly and maintenance, which is determined by characteristics of the regional species pool (Koffel et al., 2022). The regional species pool is typically assumed to be fixed; however, in hierarchical communities, the regional pool for one level of community organisation may itself be influenced by processes at a higher level of organisation. Here, we explore this idea using parasite communities, as they are tractable and highly replicable, providing an opportunity to explore the scale dependence of community assembly processes.

Key structuring forces (e.g. dispersal, ecological selection; see Vellend, 2010) may operate on multiple scales, with a series of abiotic and biotic filters that organisms need to pass through before recruiting successfully into communities (Thompson et al., 2020). Parasite ‘infracommunities’ (the collection of parasite species inside a host individual) thus allow for the assessment of the role of environmental filters across *multiple* ‘regional species pools’ (host communities and metacommunities), as well as determining the importance of fine‐scale ‘microhabitat’ factors (individual host characteristics), which may be influential in determining species distributions but are often overlooked in studies of community assembly (see Kupers et al., 2019). Parasites may be excluded from host individuals, from a host species within a site, or from whole sites (Bashey, 2015). Such parasite distribution patterns may be driven by within‐host competition (Chase & Myers, 2011; Dallas et al., 2019), the host species that are present at a given location (Mihaljevic et al., 2018) and broader characteristics of the environment in which those host species exist (Penczykowski et al., 2016). In this study we demonstrate (i) the interacting role of site‐level, host‐level and parasite‐level factors in governing the ecological assembly of parasite communities and (ii) that failing to account for patterns at a higher scale affects our ability to detect processes occurring at a lower scale.

We first consider *β*‐diversity (that is, the change in community composition among sites, Baselga, 2010) as an important concept for exploring the scale dependency of processes driving community structure. While *β*‐diversity is useful to describe, for instance, variation in parasite communities between host species (e.g. Fecchio et al., 2017), by examining relative *β*‐diversity across scales we can also compare the strength of different structuring forces (Moss et al., 2020). For example, greater *β*‐diversity between host species than between different geographic sites suggests that host specificity or selection is more important than parasite dispersal at limiting parasite recruitment to a given host individual. However, such patterns require careful consideration: the presence of a certain parasite in one host species but absence in another could be caused by host specificity, but also potentially by a low parasite prevalence meaning that the parasite was, by chance, not observed in one species. We refer to this as ‘passive sampling’ (Ulrich et al., 2009); though we note that a key premise of our study is that these prevalences themselves are driven by ecological processes at higher levels of community organisation. Few studies have previously examined parasite *β*‐diversity across scales (but see Johnson et al., 2016; Moss et al., 2020), making unclear not only the relative contribution of geographic site, host species or parasite–parasite interactions to parasite community structure but also how variables such as underlying parasite prevalence may confound analyses of this.

In this study, we also consider within‐host nestedness and how our ability to detect it is altered by whether or not we account for patterns at higher scales. Ecological nestedness describes the degree to which species‐poor communities are subsets of richer ones, and can be considered a subset of overall *β*‐diversity (Baselga, 2010). Nestedness is important to understand generally as it can have consequences for community stability and change (Yan, 2022): for example, in parasite communities, the level of nestedness may control the transmission rate of virulent pathogens (Johnson & Hoverman, 2012). The degree of nestedness in parasite community structure can be driven by factors such as differences in arrival order of parasites and subsequent priority effects (e.g. Halliday et al., 2017). However, much like *β*‐diversity, nestedness could also reflect parasite prevalence rather than direct parasite–parasite interactions with more abundant parasite species found in many hosts, and rarer parasite species found in only a subset of those hosts. Passive sampling could also be caused by characteristics of host individuals: for example, older hosts have a greater lifetime chance of accumulating parasite species (Brian & Aldridge, 2021a), which could create a nested pattern when examining a host population with a diverse age structure.

Understanding the importance of *β*‐diversity and nestedness for parasite community structure thus requires a careful partitioning of patterns caused by ecological drivers (dispersal limitation, host specificity, parasite–parasite competition) from those caused by passive sampling. However, both can be (and likely are) important across scales: for example, within‐host nestedness could be entirely caused by variable parasite prevalence at a given site (e.g. Rynkiewicz et al., 2019; =passive sampling); however, that variable prevalence may be driven by different levels of dispersal limitation in reaching that site (=ecological driver). Therefore, in this study we employ a novel model system (freshwater mussels and their parasites) to comprehensively explore the drivers of *β*‐diversity and nestedness across ecological scales, providing important and defendable evidence for the multi‐scale nature of parasite community assembly.

We analysed *β*‐diversity and nestedness across two species, three sites and two time periods. We compared our results to four different null models that vary in their constraints, from only total parasite count being constrained (least constrained null model) to both individual host infracommunity richness and parasite species prevalence being constrained (most constrained null model), allowing us to distinguish ecological drivers from passive sampling (see Methods). Given the acknowledged importance of within‐host interactions (Dallas et al., 2019), we confirmed and extended our results by implementing a Markov Random Fields model (Clark et al., 2018), which allowed us to examine the proportional impact of time, site and mussel species, as well as individual host‐level characteristics and within‐host parasite interactions. Our results demonstrate that parasite communities are structured by factors at a range of scales, from site and host community to within‐host parasite interactions. Furthermore, we show that a critical assessment of null models is both necessary and desirable to fully understand deviations from random community structure.

## 2 MATERIALS AND METHODS

### 2.1 Sampling regime

**FIGURE 1:**
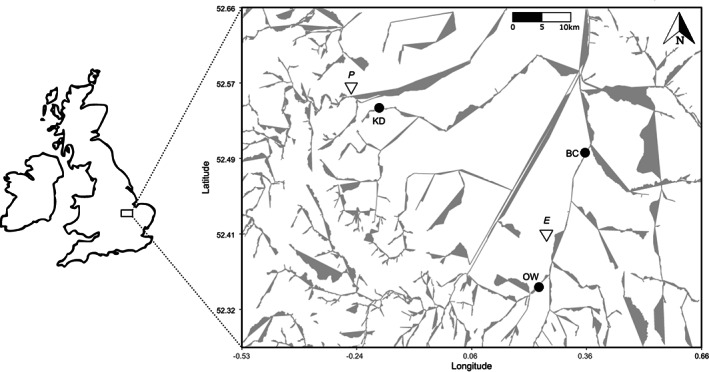
Map of the three sampling sites (black dots), where grey lines represent water bodies. BC = Brandon Creek; KD = King's Dyke; OW = Old West. For context, the population centres of Ely (E) and Peterborough (P) are also included (open triangles).

We collected mussels from three sites in Cambridgeshire, UK: Brandon Creek (henceforth BC), King's Dyke (KD) and the Old West River at Stretham (OW), all of which are part of the Great Ouse river system (Figure 1). Sampling incorporated two species: the duck mussel *Anodonta anatina* (Linnaeus 1758) and the painter's mussel *Unio pictorum* (Linnaeus 1758), both non‐endangered unionid bivalves common throughout Europe (Lopes‐Lima et al., 2017) that possess a broad range of parasites (Brian & Aldridge, 2019). We sampled on two occasions: 7 May 2019 (“Visit 1”) and 7 November 2019 (“Visit 2”).

At all sites, mussels were sampled by hand from the river margin. In total across both visits, we collected 420 mussels (240 *A. anatina*, 180 *U. pictorum*). Because extended storage of live mussels in the laboratory could cause parasites to leave the host, or move between hosts, we immediately placed mussels in 75% ethanol upon sampling before transporting them back to the laboratory. The two species were not mixed at any point once being removed from the river. Exploratory dissection on both ethanol‐stored and live mussels showed that the storage of mussels in ethanol prior to analysis did not affect the detection of any parasites in the study, and it has previously been shown as an effective way of sampling mussel parasites (Conn et al., 2008). As we only sampled non‐threatened non‐cephalised invertebrates, no ethical approval was required.

### 2.2 Mussel dissection

In the laboratory, we sliced the anterior and posterior adductor muscles to open the mussel. We inspected all parts of the mussel in systematic fashion, and identified all parasites to the finest possible taxonomic resolution using keys (we did not use molecular identification; see S1: Supplementary Methods). We inspected samples of mantle fluid (1 ml) under 40× magnification using a GXM‐L3200 compound microscope to identify the presence of ciliates and nematodes. The mantle, gills and pericardial cavity of the mussel were inspected under a GXMMZS0745‐T stereomicroscope at 16× magnification to identify further ciliates, mites, chironomids, bitterling *Rhodeus amarus* embryos and aspidogastrean trematodes. Finally, we squashed samples of gonad tissue between two glass microscope slides and studied them at 40× magnification (following Brian & Aldridge, 2020) to identify digenean trematodes. For ciliates and digenean trematodes we only noted presence or absence, while for mites, chironomids, bitterling embryos, aspidogastrean trematodes and nematodes we also counted the numbers of individuals. If the same parasite appeared in multiple life‐history stages or in multiple host tissues, we treated them separately (following Brian & Aldridge, 2021a). We measured the maximum length of all mussels (nearest 0.5 mm) with Vernier callipers, dried them to constant mass (nearest 0.001 g), and identified them as either male, non‐gravid female or gravid female via inspection of their gill tissue (where gravidity refers to the phenomenon of female unionid mussels harbouring larval mussels in specialised water‐tubes in their gills). For further details on life‐history strategy and characteristics of all parasites, see Table S1, the additional information in S1: Supplementary Results, and Brian and Aldridge (2021a). For prevalence and mean abundance for all parasites across host species and sites, see Table S2.

### 2.3 Additive and multiplicative partitioning of diversity

**FIGURE 2:**
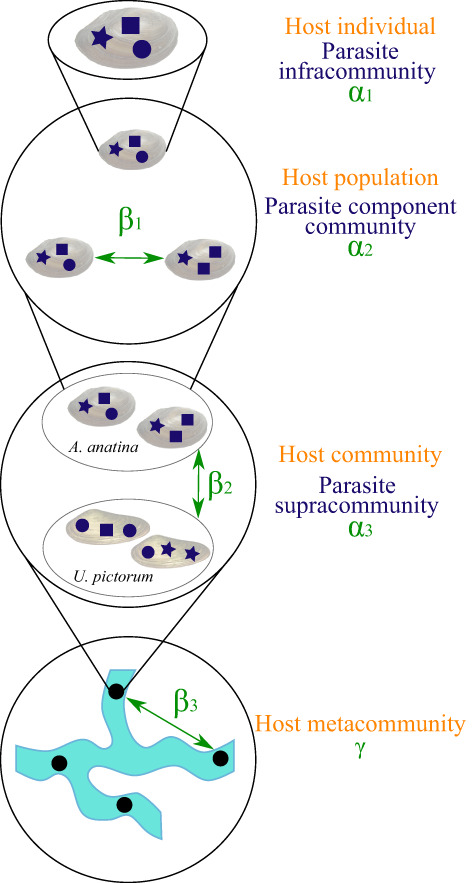
Terms used to describe host and parasite structure across scales. Terms in orange refer to host organisation; terms in blue refer to parasite organisation; terms in green refer to the division of diversity in the partitioning framework employed. Blue stars, squares and circles indicate different parasite species. Parasite terms are consistent with those of Bush et al. (1997). Figure adapted from Johnson et al. (2016).

All statistical procedures in this and the subsequent sections were executed in R v3.6.3 (R Core Team, 2020). To explore *β*‐diversity in parasite richness across different scales, we implemented an additive partitioning approach (Crist et al., 2003; Gering et al., 2003; Lande, 1996). We used this approach to explore parasite *β*‐diversity among individual hosts in a population, between the two species within a site, and among the three sample sites (Figure 2). We refer to the parasite community inside a single host organism as the infracommunity (following Bush et al., 1997). As the response variable is parasite richness, this analysis used only presence–absence data (those parasites with recordings for intensity were reduced to a present/absent recording). In this framework, *α*_1_ represents the individual parasite richness (infracommunity) of a single host within a population (with mean richness across all hosts *ᾱ*_1_), *α*_2_ represents the total parasite richness of a host population within a site (mean *ᾱ*_2_), *α*_3_ represents the total parasite richness of a given site (i.e. in the host community) (mean *ᾱ*_3_) and γ represents the total parasite diversity of the region (i.e. in the host metacommunity) (Figure 2). The relative importance of each spatial scale on parasite community structure can then be described using *β*‐diversity, such that *β*_1_ represents the average between‐host variation (within a population), *β*_2_ represents the average between‐species variation (within a site) and *β*_3_ represents the average between‐site variation, where

(1) $$\beta_{1}={ᾱ}_{2}–{ᾱ}_{1},$$

(2) $$\beta_{2}={ᾱ}_{3}–{ᾱ}_{2},$$

(3) $$\beta_{3}=\gamma –{ᾱ}_{3},$$

and, therefore,

(4) $$\gamma ={ᾱ}_{1}+\beta_{1}+\beta_{2}+\beta_{3}.$$

The adipart function in the vegan package (Oksanen et al., 2019) was used to carry out additive partitioning according to Equation 4. We carried out the additive partitioning procedure separately for Visit 1 and Visit 2. In addition, we visually explored general patterns of parasite incidence across individuals, species and sites using a two‐dimensional NMDS, also implemented in vegan using the Jaccard similarity index. The two‐dimensional ordination was sufficient to appropriately visualise the data (stress = 0.109).

In recognition of the debate concerning additive versus multiplicative partitioning for the decomposition of diversity across scales (see Jost, 2007; Veech & Crist, 2010), we also carried out multiplicative partitioning, using the multipart function according to Equation 5, where each of *β*_1_, *β*_2_ and *β*_3_ were calculated as

(5) $$\beta_{i}={ᾱ}_{i+1}/{ᾱ}_{i}$$

for *i* = 1, 2 and 3.

### 2.4 Null model analysis

To assess the significance of the obtained diversity values, we compared the results to 10,000 simulations using four different null models, which vary in the constraints they place on the null model matrix (Table 1, Figure S3). Using multiple null models can reveal important ecological trends (Crist et al., 2003; e.g. see Belmaker et al., 2008; Rynkiewicz et al., 2019). In addition, given the intense continued debate about the appropriateness of various null models (Molina & Stone, 2020), we believe it is most transparent to use and present a range of models.

**TABLE 1:** Description of the four null models used in the study. For full mathematical description, see Figure S3 (Supplementary Methods: Null model analysis in S1 Supplementary Material)

Constraint	Null model			
	EE	EF	FE	FF
Total parasite count maintained?	Yes	Yes	Yes	Yes
Prevalences of parasite species maintained?	No	Yes	No	Yes
Parasite richness inside host individuals maintained?	No	No	Yes	Yes

The EE model is the least constrained, with only the total sum of all observed parasites across all host individuals constrained to match the observed total. The EF model constrains the prevalence of parasite species to match their observed values. Therefore, if *β*‐diversity at a certain scale deviates from expectations under the EE null model, but not the EF null model, we can attribute the pattern of *β*‐diversity to parasite prevalence, and that by chance alone (due to low overall prevalence) a given parasite species was missing from a site or host species. In contrast, the FE model constrains parasite richness inside host individuals to match their observed values. This accounts for the inherent capacity of some host individuals to be more suitable (i.e. they are simply older and have had a greater chance to accumulate parasite species). The FF model constrains both parasite prevalence and infracommunity richness; therefore, patterns of *β*‐diversity that still deviate from the FF null model are strongly suggestive of ecological drivers. The EE model was implemented with the “r00” option (algorithm first developed by Atmar & Patterson, 1995), the EF model with “c0” (Jonsson, 2001), the FE model with “r0” (Patterson & Atmar, 1986) and the FF model with “quasiswap” (Miklós & Podani, 2004).

### 2.5 Nestedness

We calculated the nestedness of the observed parasite infracommunities (the tendency for species‐poor communities to be subsets of richer communities) using the measure NODF (Almeida‐Neto et al., 2008), again using only presence‐absence data. We compared the observed value to 10,000 simulations of the same four null models (EE, EF, FE, FF) using the oecosimu function in vegan. In addition, we examined patterns of nestedness separately for each visit and for each species, using the same procedure outlined above.

### 2.6 Markov Random Field modelling

We further explored the influence of visit, site, mussel species, host characteristics (length, sex, weight) and potential parasite interactions with a Conditional Random Field (CRF) analysis (Clark et al., 2018). This is an extension of a Markov Random Fields model that incorporates a matrix of covariates, and facilitates partitioning of variance in community structure among environmental factors and parasite interactions. Our covariate matrix included the factors ‘Visit’ (Visit 1, Visit 2), ‘Site’ (BC, KD, OW), ‘Species’ (*A. anatina*, *U. pictorum*), as well as the mussel‐specific characteristics ‘Length’ (in mm), ‘Sex’ (male, non‐gravid female, gravid female) and ‘Weight’ (in g, residuals of a regression of weight against length, to account for differences in weight not due to length). The continuous variables Length and Weight were scaled to have mean 0 and standard deviation 1.

To account for parameter uncertainty, we fitted a bootstrapped‐CRF to our data, using 10 bootstraps and all other default settings in the MRFcov package (Clark et al., 2018). Model performance was assessed with 10‐fold cross‐validation, with each 10‐fold training run being repeated 10 times. Combined sensitivity + specificity was 1.52, indicating good performance (Power et al., 2013). To allow direct comparison of results to our measures of *β*‐diversity and nestedness (which used presence‐absence data), we also only used presence‐absence data for our CRF. However, ignoring parasite abundance may have significant consequences on assessments of parasite community structure (Brian & Aldridge, 2021a, 2021b), and so we carried out a similar analysis using a Joint Species Distribution Model (Tikhonov et al., 2020). This framework can incorporate presence‐absence and intensity data, which allowed us to include abundances for parasites where we had such information (Table S1). For details on JSDM fitting, see S1: Supplementary Methods. Based on the results of our CRF, we carried out univariate tests on important variables to characterise the specific direction, size and significance of effects.

## 3 RESULTS

In total, there were 14 parasite groups identified (Table S1), with 2.91 ± 1.58 parasites per mussel (mean ± SD). Of the 14 parasites, two were specific to *A. anatina* and one was specific to *U. pictorum*, with 11 found in both hosts (Tables S1 and S2). All 14 parasites were found in Visit 1, while 13 were present in the Visit 2 (absence of bitterling embryos).

### 3.1 Additive partitioning and nestedness results

**FIGURE 3:**
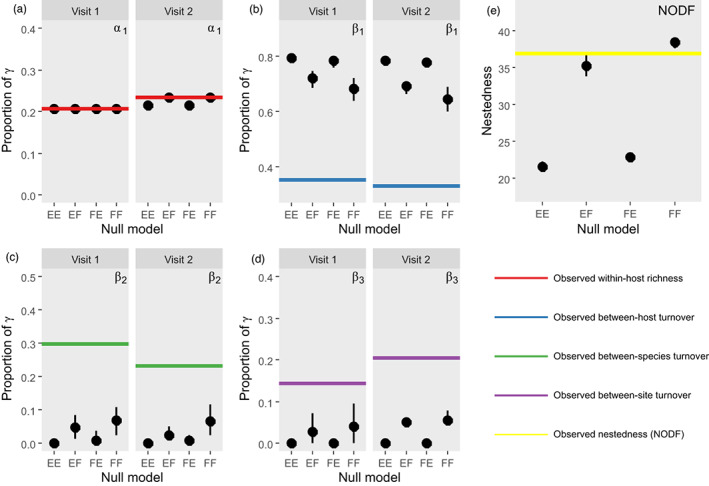
Results of additive partitioning of variance and nestedness for the observed data, and their comparison with the four null models. Actual observed values of *β*‐diversity and nestedness represented by horizontal lines. (a) ᾱ1 (red); (b) *β*1 (blue); (c) *β*2 (green); (d) *β*3 (purple); (e) NODF (yellow). The values for the 10,000 null model simulations are visualised by black dots, with error bars indicating the extent of 95% of null model values. For the additive partitioning, separate panels are presented for Visit 1 (May) and Visit 2 (November).

Additive and multiplicative partitioning yielded identical conclusions. We present the additive partitioning results here, to enable comparison with previous parasite work (Johnson et al., 2016; Moss et al., 2020). Multiplicative partitioning results are presented in S1: Supplementary Results (Table S3). Considering first the additive partitioning, regardless of the visit or the null model employed, results were consistent for the partitions *β*_1_, *β*_2_ and *β*_3_ (Figure 3). For all four null models and for both visits, the observed *β*_1_ was significantly lower than null model predictions (differences: Visit 1 EE = −0.44, FE = −0.43, EF = −0.37, FF = −0.33; Visit 2 EE = −0.45, FE = −0.45, EF = −0.36, FF = −0.32; *p* < 0.001, all cases), indicating that *β*‐diversity among individual hosts was less than what would be expected by chance. Similarly, the observed *β*_2_ and *β*_3_ values were all significantly higher than null model expectations (*β*_2_: Visit 1 EE = 0.30, FE = 0.29, EF = 0.25, FF = 0.23; Visit 2 EE = 0.23, FE = 0.22, EF = 0.21, FF = 0.16; *β*_3_: Visit 1 EE = 0.14, FE = 0.14, EF = 0.12, FF = 0.10; Visit 2 EE = 0.21, FE = 0.21, EF = 0.15, FF = 0.15; *p* < 0.001, all cases), highlighting that beta‐diversity both between host species and sites was greater than expected by chance. However, the significance of the parameter *α*_1_ was dependent on the visit and the null model employed. In Visit 1, the null model predictions were always identical, and matched the observed alpha‐diversity (*p* = 1, all cases). In Visit 2, while the EF and FF null models always matched the observed alpha‐diversity (*p* = 1 in both cases), the EE and FE null models predicted significantly lower diversity than what was observed (difference: 0.17 in both cases, *p* < 0.001).

While the choice of null model generally did not influence the overall result, there were clear differences between the four. The two null models that did not constrain parasite prevalence (EE, FE) gave very similar results, while likewise the two models that did constrain parasite prevalence (EF, FF) were highly consistent in their predictions, and closer to the observed values (Figure 3). In particular, the EE and FE models resulted almost exclusively in predicting that both *β*_2_ and *β*_3_ were equal to zero.

**FIGURE 4:**
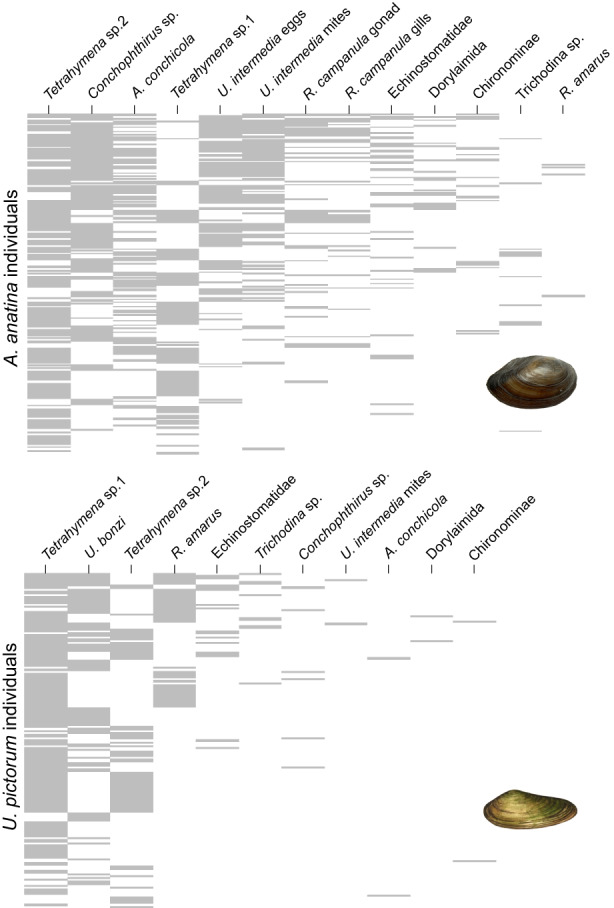
Nestedness matrices for *Anodonta anatina* (top) and *Unio pictorum* (bottom). Rows represent host individuals, columns represent parasite species or morphospecies, grey bars represent presence (i.e. a parasite species that was observed in a host individual). Rows and columns are ordered top to bottom and left to right according to total sum.

In contrast, conclusions about observed nestedness depended on null model choice. When compared with EE, EF or FE models, the observed NODF (36.9) was greater than null model predictions (differences: EE 15.37, *p* < 0.001; FE 14.07, *p* < 0.001; EF 1.60, *p* = 0.037), indicating the within‐host parasite communities (infracommunities) were more nested than expected by chance (Figure 3), a trend visible in species‐specific nestedness matrices (Figure 4). In contrast, compared with the FF null model, which constrains the parasite prevalence and individual host richness to their observed values, actual nestedness was lower than expected by chance (FF ‐1.51, *p* < 0.001; Figure 3). However, as with the *β*‐diversity results, EF and FF models yielded similar predictions, which were very close to the observed nestedness. These results were qualitatively consistent across both time periods (Table S4), and for both host species separately (Figure 4).

### 3.2 Conditional Random Field modelling

**FIGURE 5:**
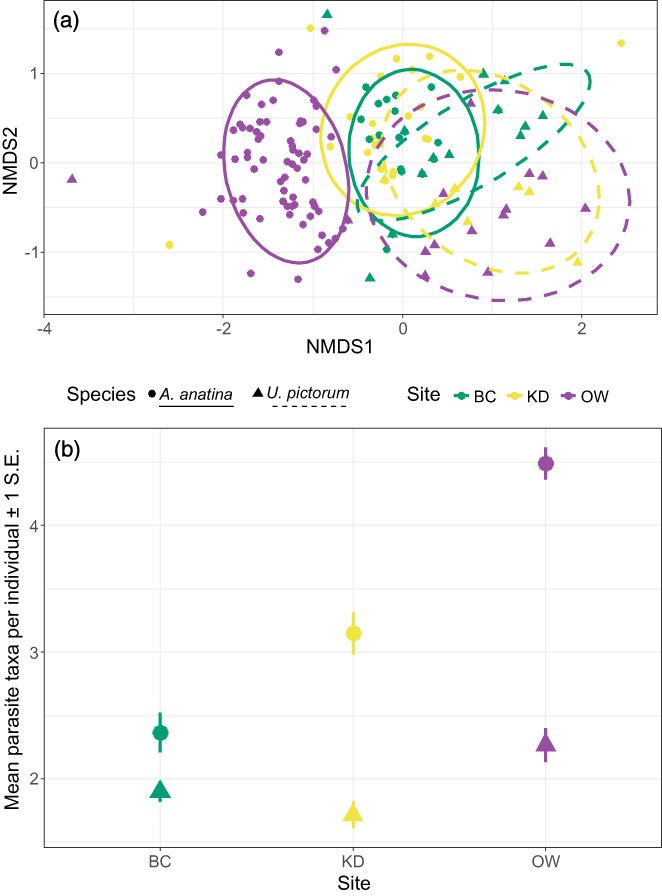
Patterns of parasite structure across host individuals, populations, species and sites. (a) NMDS visualisation, where each point represents a parasite infracommunity. Coloured circles represent sites (purple: OW; green: BC; yellow: KD), with different shapes and line types for each species (circles and solid lines, *A. anatina*; triangles and dashed lines, *U. pictorum*). (b) Mean richness, with the associated standard error, of *A. anatina* (circles) and *U. pictorum* (triangles) at each of the three sites (BC = Brandon Creek; KD = King's Dyke; OW = Old West).

CRF results support those above, particularly the additive partitioning results. Host species (*A. anatina* or *U. pictorum*) and site (BC, KD or OW) were the two most significant contributors to variation in community structure, explaining 40.5% (host species) and 25.1% (site) of the variation respectively, with clear clusters in the NMDS visualisation (Figure 5a). These results were highly consistent with those of the JSDM, which incorporated parasite abundance and fit the data well (Table S5), where 40.1% and 33.4% of the variation was explained by species and site respectively (Figure S4). *Post‐hoc* univariate tests further explored role of host species and site (Figure 5b). At each of the three sites, *A. anatina* individuals possessed a higher parasite richness than *U. pictorum* (*p* = 0.027 [BC], *p* < 0.001 [KD, OW]). However, this difference ranged from 1.3‐fold (BC) to 2‐fold (OW). This difference was not due to differences in the total number of sampled individuals of each species: rarefaction shows that for a random sample of 10 individuals of each species, there is nearly double the parasite richness in *A. anatina* (3.63 ± 0.10; mean ± S.E.) than *U. pictorum* (1.96 ± 0.07).

Considering *U. pictorum* individuals only, BC and KD had similar richness (*p* = 0.151), while OW had 1.2‐fold higher richness than BC (*p* = 0.038) and a 1.3‐fold higher richness than KD (*p* = 0.008). Considering *A. anatina* individuals only, OW had a 1.4‐fold higher richness than KD (*p* < 0.001), and a 1.9‐fold higher richness than BC (*p* < 0.001). In addition, KD also had a 1.3‐fold higher richness than BC (*p* = 0.004). Therefore, in general, individual mussels at OW had greater richness than the other two sites, but to a much greater extent in *A. anatina* than *U. pictorum* (Figure 5b). However, all three sites had a similar total parasite richness (BC = 13; KD = 11; OW = 14).

**FIGURE 6:**
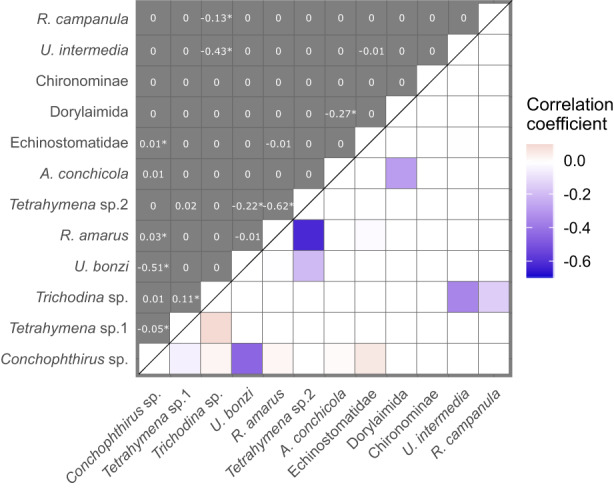
Mean correlations between parasite taxa (upper diagonals) with statistical significance denoted by (*), with significant correlations visualised as a heat map (lower diagonals).

In contrast to species and site effects, individual mussel characteristics (length, weight, sex) had little effect, collectively explaining just 4% of the parasite community variance, though there is still a large amount of variation between host individuals of the same species at the same site (Figure 5a). However, within‐host parasite interactions explained 12.3% of the variance, suggesting that competition may play a role even after taking into account site‐ and species‐specific differences (Figure 6). While the majority of interactions were neutral at the 95% confidence level, there were 10 putative interactions (Figure 6). Seven occurred between parasites occupying the same tissue of the mussel, of which five were negative (Figure 6). Of the three interactions that occurred between parasites in different tissues, two were negative. In particular, the ciliates (*Conchophthirus* sp., *Tetrahymena* sp.1 and sp.2, and *Trichodina* sp.) had a range of negative correlations with other species occupying the gills and mantle of the mussel, such as the mites *U. intermedia* and *U. bonzi* and bitterling larvae *R. amarus*.

## 4 DISCUSSION

This study compared observed *β*‐diversity and nestedness to multiple null models to explore facets of parasite community assembly across scales. We also employed a Markov Random Fields model to extend these results and focus more closely on the factors governing host infection in this system. We found that mathematical constraints placed on null models affected results. This did not affect the direction of both additive and multiplicative partitioning, which found that between‐site (*β*_3_) and between‐species (*β*_2_) diversity was much greater than expected by chance, while between‐individual diversity (*β*_1_) was lower than expected. However, null model choice affected conclusions concerning nestedness, where the most constrained null model reversed the interpretation that the parasite communities were more nested than expected by chance.

### 4.1 Null model choice affects results

With respect to *β*‐diversity (additive partitioning), the choice of null model did not affect the significance of the parameters *β*_1_, *β*_2_ and *β*_3_, but it did for ᾱ_1_ (Figure 3). This is because *α*‐diversity is dependent on γ‐diversity and γ‐diversity changed between the two visits, a nuance only able to be detected by certain null models. Because this is a more methodological point, we reserve in‐depth discussion for S1: Supplementary Discussion, although we note that given additive partitioning is a zero‐sum game, this artificially raises one or more *β*‐parameters. This result is very interesting and important, as it highlights how the time‐specificity of γ‐diversity is instead observed at the *α*‐diversity level inside this framework, with corresponding consequences for interpretations of *β*‐diversity. Despite this incongruence at the *α*‐diversity level, interpretations of *β*‐diversity were consistent regardless of which null model was used: between‐host *β*‐diversity was less than expected, while between‐species and between‐site *β*‐diversity was greater than expected (Figure 3). These results were the same for multiplicative partitioning, in terms of both *α*‐ and *β*‐diversity (Table S3).

Conversely, whether parasite infracommunities were considered significantly nested varied with null model selection. Comparisons of the observed nestedness to predictions from the EE, EF or FE null models suggest that infracommunities are significantly *more* nested than expected (Figures 3 and 4); in contrast, comparing to the FF model, with both parasite prevalence and individual host richness constrained, suggests parasite infracommunities are significantly *less* nested than expected. Taken together with the additive partitioning results, we make the following four statements: (1) *β*‐diversity in parasite communities between host species, and between sites, is larger than expected; (2) This observed *β*‐diversity cannot be explained by parasite prevalence or individual host richness; (3) Parasite infracommunties were more nested than expected, even when considering parasite prevalence by itself, or individual host richness by itself; (4) Accounting for both parasite prevalence and individual host richness simultaneously, infracommunities were less nested than expected. Below, we treat each statement in turn and consider their implications.

### 4.2 Statement 1: *β*‐diversity in parasite communities between host species, and between sites, is larger than expected

This conclusion is consistent with previous work, which has found host species and location to be major determinants of parasite community composition (e.g. Dallas et al., 2019; Vidal‐Martínez & Poulin, 2003). This trend is expanded on by Figure 5, which highlights similar parasite richness for individual *U. pictorum* hosts at all three sites, while being slightly elevated at OW. In contrast, *A. anatina* shows a clear increasing gradient from BC to OW. While overall site parasite richness was slightly higher at OW (14 parasites, vs. 11 [KD] and 13 [BC]), it is not to the same extent as the increase in individual *A. anatina* richness, suggesting that the interaction is driven by higher prevalences of parasites specific to *A. anatina* at KD and OW, such as *R. campanula*, the castrating trematode (Taskinen et al., 1997).

Competition between hosts can be mediated by parasites (Hatcher et al., 2012), something which can vary on a population level (Reichard et al., 2015). If this is the case, our results suggest there could be different outcomes of competition at BC (where *A. anatina* and *U. pictorum* individuals have similar parasite richness) and OW (where *A. anatina* individuals have on average twice as many parasites). Supporting this is the very different distributions of points between the two species at OW (purple circles) in Figure 5, to a much greater extent than at the other two sites. Given the importance of unionid mussels as ecosystem engineers (Vaughn, 2018), a distribution of parasite pressure that is uneven at both the site and host species level could have a significant effect on the wider environment, highlighting the need to consider parasite richness across sites and within host species.

### 4.3 Statement 2: This observed species and site *β*‐diversity cannot be explained by parasite prevalence or individual host richness

Even accounting for parasite prevalence or site‐specific host richness patterns, null models predicted that *β*_2_ and *β*_3_ should be ~0 (Figure 3); in other words, based on the prevalence of the different parasites in the study, and the mean infracommunity richness of all the hosts, it is statistically likely that all sites should have all parasites, and these parasites should be found in both host species. The fact that this is not observed is strongly suggestive of environmental filtering at multiple scales: parasites' dispersal ability to hosts is being mediated by both between‐site differences, and subsequently by differences between host species within sites (i.e. host compatibility).

Successful infection is reliant on propagule pressure, as well as on abiotic and biotic factors (Catford et al., 2009). Propagule pressure, in terms of parasite infective stages being present in the environment, provides a valid explanation for the different prevalences between sites within a single species (Olori et al., 2018). Many of the parasites in our study system (trematodes and mites) require multiple hosts in their life cycle, and these additional hosts may exhibit differential abundance between the sites, thus limiting the ability of the parasite to complete its life cycle (Lafferty & Harvell, 2014). Cryptic unmeasured environmental (abiotic) variation between the sites could affect the transmission stages of the parasite (Penczykowski et al., 2016), and hence also interact with propagule pressure as perceived by individual hosts.

These are plausible non‐mutually exclusive hypotheses to explain the mean richness and prevalence differences between sites (i.e. *β*_3_), but fail to account for why *A. anatina* should have significantly higher richness than *U. pictorum* at all three sites (i.e. *β*_2_) (Figure 5b): a biotic filter is clearly operating. This raises the question of primary vs. secondary unsuitability (Grim et al., 2011): does *U. pictorum* have superior host defence, or are parasites preferentially infecting *A. anatina*? While from an observational standpoint these lead to the same outcome, it has important consequences. If it is the former explanation, attempted infections of *U. pictorum* are ‘wasted’, and this could actually help *A. anatina* through a diluting effect (Rigaud et al., 2010). In contrast, if parasites specifically target *A. anatina*, their fitness may be compromised relative to *U. pictorum*, and suffer in competitive interactions. Previous research has shown the importance of co‐evolutionary history between hosts and parasites (Reichard et al., 2012); it is possible that *A. anatina* has a longer co‐evolutionary history with the suite of parasites in the community, which explains the closer association with them. While beyond the scope of the current study, the hypotheses developed here provide avenues of future research.

### 4.4 Statement 3: Parasite infracommunties were more nested than expected, even when considering parasite prevalence by itself, or individual host richness by itself

Comparisons with three of the four null models (excluding FF) suggest that low‐richness parasite infracommunities are nested subsets of richer infracommunities. Patterns of both parasite prevalence (Rynkiewicz et al., 2019) and richness in individual hosts (Poulin & Valtonen, 2001) can contribute to such nestedness, and it appears both are acting here.

Differential prevalences in a parasite community cause nestedness in intuitive fashion: assuming equivalent dispersal ability, highly prevalent parasites are likely to be found in many hosts, with progressively lower‐prevalence parasites found in subsets of those hosts. Parasite prevalence is clearly contributing to nested patterns: allowing it to vary randomly led to extremely low nestedness, while constraining it to the observed values led to much higher predicted nestedness (compare NODF of EE and EF models, Figure 3). In contrast, the EF and FF models were quite similar, and very close to the observed nestedness (despite slight statistical differences in both cases). Ecologically, this suggests that parasite prevalence (the constraint shared between EF and FF) at the site level explains most of the nestedness at the within host level. Drivers of dispersal from the regional species pool to given sites, and environmental conditions determining the survival of propagules at those sites, collectively determine site‐level prevalence. Going forward, these should be considered as key factors determining the within‐host structure of parasite communities, as well as site‐ and host species‐level patterns (Statement 2).

Individual host richness did still contribute to nestedness to a lesser extent. Taking into account the observed host richness increased nestedness slightly (compare NODF of EE and FE models, Figure 3) suggesting hosts with richer infracommunities were more likely to have parasites consistently absent from smaller infracommunities. This pattern can be caused by a range of factors (Baselga, 2010) that are not immediately distinguishable in an observational context. However, host characteristics such as size have previously been shown to generate nestedness (Vidal‐Martínez & Poulin, 2003). Size reflects greater consumptive ability (= sampling ability of parasites in the environment) or age, which make progressively larger or older organisms more likely to host rarer parasites (see Brian & Aldridge, 2021a), thereby producing a pattern where the infracommunities of younger or smaller hosts are predictable subsets of larger ones. We suggest that host characteristics do have a role to play in our study system: while between‐host diversity (*β*_1_) was less than expected by chance, it still provided the single biggest contribution to γ‐diversity in the study (~34% of γ; Figures 3 and 5a), and recent work has shown that, within a single site, mussel length and gravidity are both important in the construction of parasite infracommunities (Brian & Aldridge, 2021a). While the influence of site and species outweigh individual host characteristics (Figure S4), their contribution should not necessarily be considered unimportant in the context of community assembly across scales.

Freshwater mussel parasite communities are clearly nested, with implications for parasite transmission and community stability (Johnson & Hoverman, 2012; Yan, 2022). Both parasite prevalence and host richness contributed to the observed nestedness, but the similarity between the EF and FF models suggests that parasite prevalence, rather than host‐level characteristics, was a key driver of this nestedness. Nestedness was still greater than expected when considering these two factors in isolation. However, after accounting for both of these factors together (model FF), infracommunities were less nested than expected by chance (Statement 4).

### 4.5 Statement 4: Accounting for both parasite prevalence and individual host richness simultaneously, infracommunities were less nested than expected

A pattern of anti‐nestedness indicates that parasites are more dispersed than random among infracommunities (Poulin & Guégan, 2000); in other words, infracommunities are more likely to be composed of discrete modules. In our study, this pattern was observed for both host species separately, which implies that this modularity is not solely caused by communities assembling differently in the two host species. Instead, it could reflect within‐host parasite interactions (Figure 6; see Bashey, 2015; Clay et al., 2019). 70% of the non‐neutral interactions were negative, and a majority of them occurred within the same host tissue (e.g. where both parasites were in the gills of the mussel), which aligns closely with previous work that showed predominantly negative interactions between parasites occupying the same tissue (Dallas et al., 2019). Furthermore, interactions in this study system have been shown to be predominantly exclusionary (i.e. they limit the presence of parasites inside hosts, rather than their abundance; Brian & Aldridge, 2021a), which also supports the argument that competitive within‐host interactions contribute to infracommunity modularity.

We do note that the FF null model was very similar to the observed data (and for Visit 1 by itself it was not statistically different; Table S4), and so ecological interpretations of this modularity should be interpreted with caution. In particular, co‐occurrence patterns are not sufficient evidence for ecological interactions, and the correlations could be driven by an unmeasured environmental factor (Blanchet et al., 2020). Furthermore, population genetic structure can also affect infection rates through differences in defences between individuals, which could affect observed co‐occurrences in structured fashion (Sallinen et al., 2020). Competition is therefore not the only explanation for the negative co‐occurrences of parasite species, though recent experimental evidence from this system shows the presence of one parasite can inhibit the infection of another (Brian et al., 2022).

Putative modularity is only detected once parasite prevalence and host infracommunity richness are accounted for, demonstrating the importance of null model selection and accounting for different drivers occurring at different levels. Figure 4 emphasises the importance of this approach: for example, *Tetrahymena* sp.2 rarely co‐occurs with either *R. amarus* or *U. bonzi* (especially visible in *U. pictorum*) reflecting possible competitive interactions (Figure 6). A similar trend can be seen between *U. intermedia* and *Tetrahymena* sp.1 in *A. anatina* (Figure 4); however, in this case it is due to *U. intermedia* being present mainly at OW while *Tetrahymena* sp.1 is present at BC (Table S2). Therefore, parasite prevalence at the site level alters the interpretation of the interaction between *U. intermedia* and *Tetrahymena* sp. 1. Understanding *β*‐diversity at the between‐site level is important to interpret patterns at the within‐host level.

## 5 CONCLUSIONS

Community composition is significantly driven by factors at all scales of organisation (Figure 3); in this case, from between‐site and between‐species (Figure 5b) to within‐host (Figures 5a and 6). First, environmental filtering affects the presence and prevalence of certain parasites at our sites. Given the hydrological connectedness of our sites (Figure 1), we suggest that dispersal limitation is low. Instead, fine‐scale environmental differences between the sites and the distribution of host species for other stages in the life‐cycle of the parasites are likely highly significant. Future work should examine their relative roles of these factors in parasite community assembly. Next, biotic filtering in terms of host specificity influences the composition of parasite communities inside different host species. Whether this is due to choice by the parasite or defence from the host is unclear. However, our use of multiple null models confirms that the absence of certain parasites from hosts cannot be explained by low parasite prevalence and provides robust evidence for biotic filtering. In contrast, the nested structure of parasite infracommunities is largely caused by differential parasite prevalence. Subsequently accounting for prevalence and host‐level characteristics allows a possible signal of competition to be detected, with some parasites potentially limiting the successful infection of others.

Our work aligns with that of Moss et al. (2020), who also showed that parasite *β*‐diversity between host species and sites was greater than expected, while *β*‐diversity between individuals was smaller than expected. This may represent a general trend in parasite community ecology, and point towards ubiquitous drivers in community assembly which should be explored in a greater variety of systems. This conclusion also has relevance for applications such as host and parasite conservation. Given the species‐ and site dependency of parasitism, ignoring drivers of parasite community structure may jeopardise conservation action, especially for interventions such as captive breeding or translocation (Brian et al., 2021).

By distinguishing ecological drivers from passive sampling in producing the observed patterns, we extend previous work to reveal how processes influencing community structure at one level may influence the interpretation of drivers at another. The similarity between the EF and FF models that constrain prevalence, and their greater concordance with the observed results, suggest that the drivers of parasite prevalence in particular need to be carefully considered in studies of parasite community structure. These results therefore further emphasise the need to understand what drives inherent commonness and rarity in populations (Clay et al., 2019), how propagule pressure (i.e. dispersal) varies with scale, and the transition from initial entry into communities to establishment. Considering the theory of island biogeography and incidence functions which incorporate explicit spatial distances between patches/islands (Hanski, 1994) could be a valuable extension to this work. In aquatic environments, factors like river direction and flow would also be important to consider in the context of ‘island’ biogeography. This would be especially interesting over multiple spatial scales, where islands could be sites, or host individuals within sites.

## AUTHOR CONTRIBUTIONS

Both authors conceived the idea and carried out sampling. Joshua I. Brian carried out lab work, analysed and interpreted the data and drafted the manuscript. Both authors contributed to later revisions of the manuscript and gave final approval for publication.

## CONFLICT OF INTEREST

Both authors declare no conflicts of interest.

## Supporting information


Data S1.

## Data Availability

Data are available from the Dryad Digital Repository, https://doi.org/10.5061/dryad.tb2rbp047 (Brian & Aldridge, 2022a). Associated code is available at Zenodo, https://doi.org/10.5281/zenodo.7333325 (Brian & Aldridge, 2022b).
