# Supplementary material for: Factors at multiple scales drive parasite community structure
Source: J Anim Ecol. 2022 Nov 29;92(2):377–90. doi: 10.1111/1365-2656.13853 (PMC10098736; doi:10.1111/1365-2656.13853)
Supplement: Supplementary file 1 — Data S1. [file JANE-92-377-s001.docx]

**Factors at multiple scales drive parasite community structure**

**Joshua I. Brian, David C. Aldridge**

**S1 Supplementary Material**

*Contents*

| **Section** | **Page** |
| --- | --- |
| Supplementary Methods | 2 |
| Supplementary Results | 6 |
| Supplementary Discussion | 10 |
| Supplementary References | 11 |

**Supplementary Methods**

*Supplementary Methods: Parasite identification*

Most of the parasites in the present study have already been identified in freshwater mussels, and our identification was based on these previous studies (especially Brian & Aldridge 2021). This includes:

- *Conchophthirus* sp.
- *U. intermedia* (mites and eggs)
- *R. amarus*
- *Tetrahymena* sp.2
- *A. conchicola*
- *R. campanula* (in both the gill and gonad)
- Dorylaimida
- Chironominae
- Echinostomatidae (note that the ethanol storage occasionally made it difficult to make out fine-scale features of the metacercariae in the gonad and therefore discrimination to species level was not possible in the present study – to be conservative, all trematode metacercariae were given the classification of Echinostomatidae).

The parasites below were identified for the first time in the present study.

The ciliates *Trichodina* sp. were commonly observed in the mantle (Fig. S1).


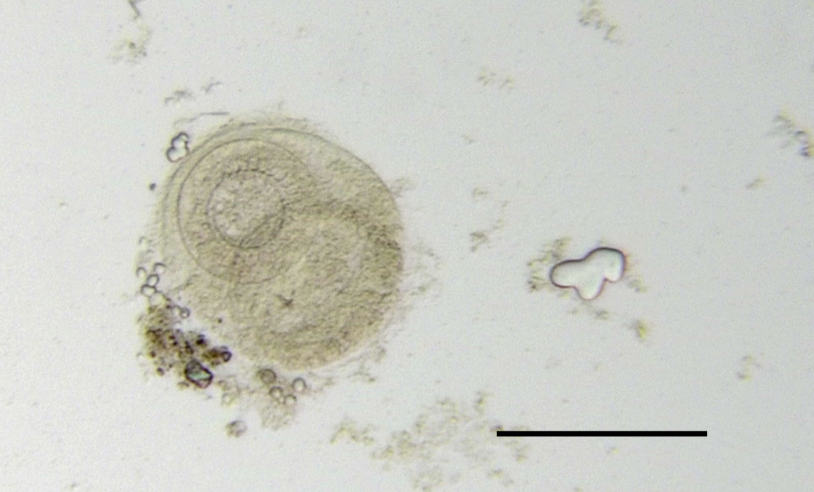


Figure S1: *Trichodina* sp. Scale bar 250 µM.

This was identified as genus *Trichodina* through consultation with Irwin et al. (2017) and Wiroonpan and Purivirojkul (2019). There is the possibility of this being *T. unionis*, given it has previously been observed as common within the hosts in this study (Fenchel 1965). However, given the absence of clear keys it has been conservatively identified as *Trichodina* sp.

The ciliates *Tetrahymena* sp.1 were also commonly observed in the mantle (Fig. S2).


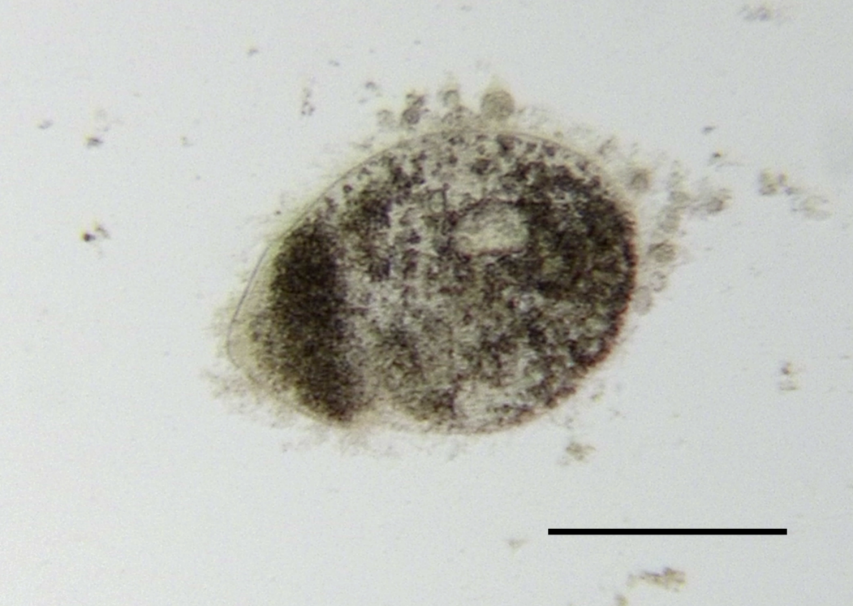


Figure S2: *Tetrahymena* sp.1. Scale bar 250 µM.

Identification of the genus *Tetrahymena* was done with reference to Martins et al. (2015) and Lynn et al. (2018). A lack of clear keys restricted the identification to genus only.

Finally, the mite *Unionicola bonzi* was identified through the ecological and descriptive information available in Davids (1973).

In two cases (*U. intermedia*, *R. campanula*) there are two separate entries in the parasite matrix. This is because they occurred in two different life-history stages (eggs and larval mites, *U. intermedia*) or in two distinct host tissues (gills and gonad, *R. campanula*). These occurrence of these entries did not appear to be obligate (e.g. there were instances in which *R. campanula* was present in the gills but not the gonad, instances in which it was present in the gonad but not the gills, and instances where it appeared in both). We argue that in both cases, the two forms cannot necessarily be assumed to be equivalent on their effects on the host, and therefore we have included both forms as separate entries in our parasite-host incidence matrix.

We did not attempt a molecular identification of parasites, either through eDNA or traditional barcoding methods. While genetic information now exists for some freshwater mussel parasites, particularly trematodes and mites (e.g. Edwards et al. 2010; Petkevičiūtė et al. 2014), their chronic under-studying (Brian & Aldridge 2019, 2022) means that most parasites lack genetic information. As such, DNA analysis would be unlikely to helpfully identity any parasites that may have been missed, as there is no suitable database to cross-reference any obtained sequences with. We do recommend that this is an urgent area of future research.

*Supplementary Methods: Null model analysis*

*
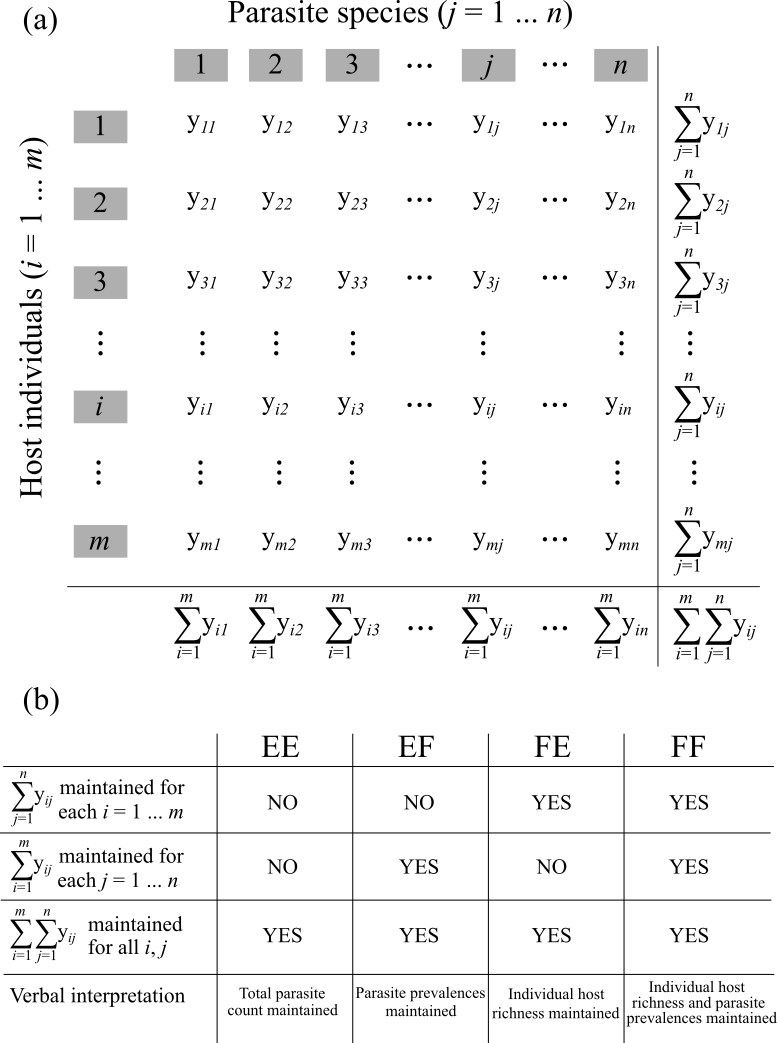
*

Figure S3: Mathematical description of the null models employed in the study. (a) The host-parasite incidence matrix, where each yij is limited to either 0 (absence) or 1 (presence). (b) Table showing the constraints on the four null models. Note that all four models also operate on the limitation that each yij can only take the values of 0 or 1. Null model names follow Ulrich and Gotelli (2007), where ‘E’ = equiprobable and ‘F’ = fixed. Therefore, EE is the least constrained (only overall sum matches observed matrix) and FF the most constrained (row totals and column totals both match observed matrix), while EF and FE are equally constrained but in opposite fashion (EF has column totals matching the observed matrix, FE has row totals matching the observed matrix).

*Supplementary Methods: Joint Species Distribution Modelling*

To further investigate drivers of community structure, we utilised a joint species distribution model, using the Hmsc package (Tikhonov et al. 2019). This uses a Bayesian framework to predict not only an individual’s response to environmental space, but also species-species interactions after accounting for all environmental variables. As this package can accept both presence-absence and quantitative data in the same procedure, we used the intensity of infection where it was available. The same six variables as for the CRF (Visit, Site, Species, Length, Weight, Sex; see *Methods* in main paper) were included in the model.

The model was constructed with the default Hmsc priors (Tikhonov et al. 2019) using 750,000 samples for each of 2 MCMC chains, with the first 250,000 samples discarded as burn-in and the remainder thinned to every 500^th^ sample. Parasites for which intensity data was available were modelled with a lognormal Poisson distribution, while parasites that only had presence-absence data available were modelled using probit regression. We confirmed convergence of beta and omega parameters, and further assessed model performance through four-fold cross-validation. Model performance was excellent (Table S5), suggesting predictions were reliable. We partitioned variance in the host parasite communities among the six explanatory variables.

*Supplementary Methods: Explanation of data files*

There are four supplementary data files accompanying this manuscript:

1. supplementary_data1.csv
2. supplementary_data2.csv
3. supplementary_data3.csv
4. supplementary_data4.csv

File 1 is the matrix of environmental and mussel traits. File 2 is the raw parasite matrix. Files 3 and 4 correspond to summarized outputs from the additive partitioning and nestedness results, and are used to reconstruct Figure 3.

**Supplementary Results**

Table S1: All parasites found in the study, including a general description of life-history and the location they occupy in the host. Host species refers to the present study only, and does not preclude the occurrence of these parasites in other host species. Presence/absence indicates that only the occurrence of a parasite in a host was recorded, while Intensity indicates that the numbers of that parasite per host were recorded.

| **Parasite** | **General description and location in host** | **Host species** | **Presence/absence or intensity** |
| --- | --- | --- | --- |
| *Conchophthirus* sp. | Ciliate that lives in the mantle; particularly associated with labial palps | Both | Presence/absence |
| *Tetrahymena* sp.1 | Ciliate that lives in the mantle | Both | Presence/absence |
| *Trichodina* sp. | Ciliate that lives in the mantle | Both | Presence/absence |
| *Unionicola intermedia* (mites) | Larval mite that encysts in the mantle | Both (but very rarely in *U. pictorum*) | Intensity |
| *Unionicola intermedia* (eggs) | Mite eggs that also encyst in the mantle | Both (but very rarely in *U. pictorum*) | Intensity |
| *Unionicola bonzi* | Mite that occupy the gills | *U. pictorum* only | Intensity |
| *Rhodeus amarus* embryos | Embryos of bitterling fish that are deposited into the gills of the host mussel | Both | Intensity |
| *Tetrahymena* sp.2 | Ciliate that lives in the gills | Both | Presence/absence |
| *Aspidogaster conchicola* | Aspidogastrean trematode that lives in the mantle, particularly under the pericardial cavity | Both (but very rarely in *U. pictorum*) | Intensity |
| Echinostomatidae | Echinostomatid trematode that encysts in the gonad as metacercariae | Both | Intensity |
| *Rhipidocotyle campanula* (gills) | Bucephalid trematode that produces long sporocysts running transversely through the host gills | *A. anatina* only | Presence/absence |
| *Rhipidocotyle campanula* (gonad) | Bucephalid trematode that produces long sporocysts and cercariae that occupies the gonad and castrates the host | *A. anatina* only | Presence/absence |
| Dorylaimida | Nematode that lives in the mantle | Both | Intensity |
| Chironominae | Chironomid larvae that live in the mantle | Both | Intensity |

Table S2: Prevalence and mean abundance of each parasite in the study, separated by host species and site. Prevalence represents the percent of individuals that had a given parasite. Intensity is calculated only from infected hosts (dashes indicate where only prevalence was measured; see Table S1).

|  | **BC** | | **KD** | | **OW** | |
| --- | --- | --- | --- | --- | --- | --- |
|  | ***A. anatina*** | ***U. pictorum*** | ***A. anatina*** | ***U. pictorum*** | ***A. anatina*** | ***U. pictorum*** |
| **Parasite species** | **Prevalence % (mean intensity)** | **Prevalence (mean intensity)** | **Prevalence (mean intensity)** | **Prevalence (mean intensity)** | **Prevalence (mean intensity)** | **Prevalence (mean intensity)** |
| *Conchophthirus* sp. | 3.3 (-) | 3.3 (-) | 16.7 (-) | 3.3 (-) | 95.0 (-) | 3.3 (-) |
| *Tetrahymena* sp.1 | 73.3 (-) | 88.3 (-) | 85.0 (-) | 81.7 (-) | 0 | 63.3 (-) |
| *Trichodina* sp. | 6.7 (-) | 5.0 (-) | 15.0 (-) | 3.3 (-) | 0 | 6.7 (-) |
| *Unionicola intermedia* (mites) | 1.7 (1.0) | 0 | 10.0 (2.5) | 1.7 (1) | 66.7 (13.1) | 1.7 (1) |
| *Unionicola intermedia* (eggs) | 5.0 (3.0) | 0 | 3.3 (3.5) | 0 | 74.2 (21.2) | 0 |
| *Unionicola bonzi* | 0 | 0 | 0 | 48.3 (2.6) | 0 | 70.0 (26.1) |
| *Rhodeus amarus* embryos | 0 | 36.7 (5.7) | 0 | 0 | 4.2 (1.6) | 33.3 (8.8) |
| *Tetrahymena* sp.2 | 90.0 (-) | 45.0 (-) | 76.7 (-) | 30.0 (-) | 64.2 (-) | 26.7 (-) |
| *Aspidogaster conchicola* | 31.7 (1.3) | 3.3 (5.5) | 51.7 (2.2) | 0 | 47.5 (1.5) | 0 |
| Echinostomatidae | 13.3 (1.9) | 5.0 (1.3) | 1.7 (1.0) | 3.3 (1.5) | 23.3 (3.1) | 18.3 (1.5) |
| *Rhipidocotyle campanula* (gills) | 5.0 (-) | 0 | 25.0 (-) | 0 | 17.5 (-) | 0 |
| *Rhipidocotyle campanula* (gonad) | 6.7 (-) | 0 | 30.0 (-) | 0 | 22.5 (-) | 0 |
| Dorylaimida | 0 | 1.7 (1.0) | 0 | 0 | 18.3 (1.2) | 1.7 (1) |
| Chironominae | 0 | 1.7 (1.0) | 0 | 0 | 15.8 (1.2) | 1.7 (1) |

Table S3: Results of multiplicative partitioning.

| **Diversity measure** | **Visit** | **Actual value** | **Null model** | **95% null model confidence interval** | **p-value** |
| --- | --- | --- | --- | --- | --- |
| α_1_ | 1 | 2.898 | EE | 2.912 – 2.961 | <0.001 |
| α_1_ | 2 | 3.024 | EE | 3.038 – 3.082 | 0.002 |
| α_1_ | 1 | 2.898 | EF | 2.907 – 2.946 | 0.003 |
| α_1_ | 2 | 3.024 | EF | 3.029 – 3.063 | 0.036 |
| α_1_ | 1 | 2.898 | FE | 2.898 – 2.898 | 1 |
| α_1_ | 2 | 3.024 | FE | 3.024 – 3.024 | 1 |
| α_1_ | 1 | 2.898 | FF | 2.898 – 2.898 | 1 |
| α_1_ | 2 | 3.024 | FF | 3.024 – 3.024 | 1 |
| β_1_ | 1 | 1.813 | EE | 4.401 – 4.651 | <0.001 |
| β_1_ | 2 | 1.895 | EE | 4.255 – 4.478 | <0.001 |
| β_1_ | 1 | 1.813 | EF | 3.393 – 3.579 | <0.001 |
| β_1_ | 2 | 1.895 | EF | 2.939 – 3.080 | <0.001 |
| β_1_ | 1 | 1.813 | FE | 5.098 – 5.465 | <0.001 |
| β_1_ | 2 | 1.895 | FE | 5.065 – 5.410 | <0.001 |
| β_1_ | 1 | 1.813 | FF | 3.691 – 4.083 | <0.001 |
| β_1_ | 2 | 1.895 | FF | 3.105 – 3.427 | <0.001 |
| β_2_ | 1 | 1.640 | EE | 1.019 – 1.049 | <0.001 |
| β_2_ | 2 | 1.550 | EE | 1.017 – 1.045 | <0.001 |
| β_2_ | 1 | 1.640 | EF | 1.018 – 1.054 | <0.001 |
| β_2_ | 2 | 1.550 | EF | 1.012 – 1.046 | <0.001 |
| β_2_ | 1 | 1.640 | FE | 1.028 – 1.075 | <0.001 |
| β_2_ | 2 | 1.550 | FE | 1.029 – 1.076 | <0.001 |
| β_2_ | 1 | 1.640 | FF | 1.038 – 1.095 | <0.001 |
| β_2_ | 2 | 1.550 | FF | 1.066 – 1.132 | <0.001 |
| β_3_ | 1 | 1.419 | EE | 1.010 – 1.031 | <0.001 |
| β_3_ | 2 | 1.347 | EE | 1.009 – 1.029 | <0.001 |
| β_3_ | 1 | 1.419 | EF | 1.007 – 1.036 | <0.001 |
| β_3_ | 2 | 1.347 | EF | 1.005 – 1.016 | <0.001 |
| β_3_ | 1 | 1.419 | FE | 1.013 – 1.044 | <0.001 |
| β_3_ | 2 | 1.347 | FE | 1.011 – 1.038 | <0.001 |
| β_3_ | 1 | 1.419 | FF | 1.023 – 1.079 | <0.001 |
| β_3_ | 2 | 1.347 | FF | 1.032 – 1.079 | <0.001 |

Table S4: Results of nestedness analysis separated by time period. Note qualitatively identical results to the overall analysis, though nestedness in time period 1 does not statistically differ from random expectations under the EF and FF models.

| **Nestedness** | **Visit** | **Actual value** | **Null model** | **95% null model confidence interval** | **p-value** |
| --- | --- | --- | --- | --- | --- |
| NODF | 1 | 33.562 | EE | 19.773 – 21.923 | <0.001 |
| NODF | 2 | 42.865 | EE | 21.138 – 23.375 | <0.001 |
| NODF | 1 | 33.562 | EF | 30.545 – 34.484 | 0.3065 |
| NODF | 2 | 42.865 | EF | 37.911 – 42.770 | 0.040 |
| NODF | 1 | 33.562 | FE | 21.338 – 22.111 | <0.001 |
| NODF | 2 | 42.865 | FE | 24.010 – 24.455 | <0.001 |
| NODF | 1 | 33.562 | FF | 33.421 – 35.291 | 0.093 |
| NODF | 2 | 42.865 | FF | 44.069 – 46.271 | <0.001 |

Table S5: Summary of Hmsc model performance. Tjur’s R^2^ measures the difference between mean fitted values between presences and absences; therefore a value and confidence limits >0 suggests the model performs significantly better than chance. AUC (Area Under the Curve) indicates the likelihood of successfully predicting a presence or an absence for a given parasite in a given host; therefore a value and confidence limits >0.5 suggests the model performs significantly better than chance at predicting presences and absences. The standard measures (first two rows, PA) are appropriate for parasites that only have presence/absence data; the next two rows (IN) provide the equivalent metric for intensity data. The final row refers to how well the model ranks abundances, can be interpreted as a traditional R^2^, and is only used for intensity data.

| **Performance metric** | **Mean** | **Standard deviation** |
| --- | --- | --- |
| PA.Tjur’s R^2^ | 0.233 | 0.200 |
| PA.AUC | 0.822 | 0.057 |
| IN.Tjur’s R^2^ | 0.449 | 0.211 |
| IN.AUC | 0.884 | 0.084 |
| SR^2^ | 0.460 | 0.186 |


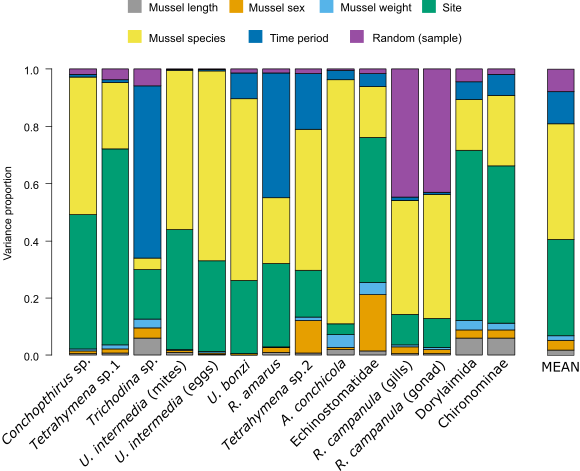


Figure S4: Variance partitioning results from the joint species distribution model. The total variation in presence/abundance of each parasite is partitioned among the six explanatory variables, in addition to random variation not attributable to any explanatory variable (purple). Overall mean is presented on far right.

**Supplementary Discussion**

*Changes in α-diversity significance with different null models*

With respect to β-diversity (additive partitioning), the choice of null model did not affect the significance of the parameters β_1_, β_2_ and β_3_, but it did for ᾱ_1_ (Fig. 3). The reason for this was a time-specific difference in parasite richness, with the parasite *R. amarus* not observed in Visit 2 (i.e. γ_Visit1_ = γ_Global_, but γ_Visit2_ < γ_Global_). Because ᾱ_1_ is expressed as a proportion of γ, in the null models each individual α_1_ equals the randomized richness of that particular host, divided by the total parasite count. Therefore, as the total parasite count is maintained for all four null models (Table 1, main text), the simulated ᾱ_1_ value should always match the observed data (i.e. while the α_1_ of individual hosts will vary among simulations, their mean will always be consistent). However, in Visit 2, those null models that do not constrain parasite prevalences to the observed values fail to account for the fact that *R. amarus* is absent (prevalence = 0), and therefore the γ value for that visit is inflated relative to the actual data. This artificially lowers ᾱ_1_ relative to the observed data for the models EE and FE (Fig. 3, main text). Because additive partitioning is a zero-sum game, this in turn will artificially raise one or more β-parameters. This result is very interesting, as it highlights how the time-specificity of γ-diversity is instead observed at the α-diversity level inside this framework, with corresponding consequences for interpretations of β-diversity. This is consistent with previous work, which has shown that inferred differences in community richness and β-diversity are driven purely by γ-diversity differences (Kraft et al. 2011). While these differences can be accounted for in the current study, in studies with more time points, greater data complexity, or that only use one null model, one might state with confidence that within-host diversity was significantly larger than expected. This could lead, for instance, to inferences of parasite facilitation inside a host, when it is instead driven by the opposite end of the hierarchical scale (Chase & Myers 2011).

**Supplementary References**

Brian, J. I., & Aldridge, D. C. (2019). Endosymbionts: An overlooked threat in the conservation of freshwater mussels?. *Biological Conservation*, **237**, 155-165.

Brian, J. I, & Aldridge, D. C (2021). Abundance data applied to a novel model invertebrate host shed new light on parasite community assembly in nature. *Journal of Animal Ecology*, **90**, 1096-1108.

Brian, J. I., & Aldridge, D. C. (2022) Mussel parasite richness and risk of extinction. *Conservation Biology*, e13979.

Bush, A. O., Lafferty, K. D., Lotz, J. M., & Shostak, A. W. (1997). Parasitology meets ecology on its own terms: Margolis et al. revisited. *The Journal of Parasitology*, **83**, 575-583.

Chase, J. M., & Myers, J. A. (2011). Disentangling the importance of ecological niches from stochastic processes across scales. *Philosophical transactions of the Royal Society B: Biological sciences*, **366**, 2351-2363.

Davids, C. (1973). The relations between mites of the genus *Unionicola* and the mussels *Anodonta* and *Unio*. *Hydrobiologia*, **41**, 37-44.

Edwards, D. D., Vidrine, M. F., & Ernsting, B. R. (2010). Phylogenetic relationships among Unionicola (Acari: Unionicolidae) mussel-mites of North America based on mitochondrial cytochrome oxidase I sequences. *Zootaxa*, **2537**, 47-57.

Fenchel, T. (1965). Ciliates from Scandinavian molluscs. *Ophelia*, **2,** 71–174.

Irwin, N. A., Sabetrasekh, M., & Lynn, D. H. (2017). Diversification and Phylogenetics of Mobilid Peritrichs (Ciliophora) with Description of *Urceolaria parakorschelti* sp. nov. *Protist*, **168**, 481-493.

Kraft, N. J., Comita, L. S., Chase, J. M., Sanders, N. J., Swenson, N. G., Crist, T. O., ... & Cornell, H. V. (2011). Disentangling the drivers of β diversity along latitudinal and elevational gradients. *Science*, **333**, 1755-1758.

Lynn, D. H., Doerder, F. P., Gillis, P. L., & Prosser, R. S. (2018). *Tetrahymena glochidiophila* n. sp., a new species of *Tetrahymena* (Ciliophora) that causes mortality to glochidia larvae of freshwater mussels (Bivalvia). *Diseases of Aquatic Organisms*, **127**, 125-136.

Martins, M. L., Cardoso, L., Marchiori, N., & Benites de Pádua, S. (2015). Protozoan infections in farmed fish from Brazil: diagnosis and pathogenesis. *Revista Brasileira de Parasitologia Veterinária*, **24**, 1-20.

Petkevičiūtė, R., Stunžėnas, V., & Stanevičiūtė, G. (2014). Differentiation of European freshwater bucephalids (Digenea: Bucephalidae) based on karyotypes and DNA sequences. *Systematic Parasitology*, **87**, 199-212.

Tikhonov, G., Opedal, Ø. H., Abrego, N., Lehikoinen, A., de Jonge, M. M., Oksanen, J., & Ovaskainen, O. (2020). Joint species distribution modelling with the R‐package Hmsc. *Methods in Ecology and Evolution*, **11**, 442-447.

Wiroonpan, P., & Purivirojkul, W. (2019). New record of *Trichodina unionis* (Ciliophora, Trichodinidae) from freshwater gastropods in Bangkok, Thailand. *Parasite*, **26**, 47.
